# Effects of Supplementation with Microalgae Extract from *Phaeodactylum tricornutum* (Mi136) to Support Benefits from a Weight Management Intervention in Overweight Women

**DOI:** 10.3390/nu16070990

**Published:** 2024-03-28

**Authors:** Broderick Dickerson, Jonathan Maury, Victoria Jenkins, Kay Nottingham, Dante Xing, Drew E. Gonzalez, Megan Leonard, Jacob Kendra, Joungbo Ko, Choongsung Yoo, Sarah Johnson, Rémi Pradelles, Martin Purpura, Ralf Jäger, Ryan Sowinski, Christopher J. Rasmussen, Richard B. Kreider

**Affiliations:** 1 Exercise & Sport Nutrition Laboratory, Department of Kinesiology and Sports Management, Texas A&M University, College Station, TX 77843, USA; dickersobl5@email.tamu.edu (B.D.); victoria.jenkins@tamu.edu (V.J.); kirsten.nottingham@cpcmed.org (K.N.); dantexing@tamu.edu (D.X.); dg18@tamu.edu (D.E.G.); meganleonard10@tamu.edu (M.L.); jkendra@tamu.edu (J.K.); joungboko10@tamu.edu (J.K.); choongsungyoo@tamu.edu (C.Y.); sjohnson2216@tamu.edu (S.J.); rjs370@tamu.edu (R.S.); crasmussen@tamu.edu (C.J.R.); 2 Research & Development Department, Microphyt, 34670 Baillargues, France; jonathan.maury@microphyt.eu (J.M.); remi.pradelles@microphyt.eu (R.P.); 3 Increnovo LLC, Whitefish Bay, WI 53217, USA; martin.purpura@increnovo.com (M.P.); ralf.jaeger@increnovo.com (R.J.)

**Keywords:** fucoxanthin, obesity, appetite, bone mineral content, bone density, aerobic capacity, inflammation, blood lipids, functional capacity, quality of life

## Abstract

Background: Microalgae like *Phaeodactylum tricornutum* (PT) contain the carotenoid, fucoxanthin, which has been purported to promote fat loss, lower blood lipids, and improve glucose management. This study examined whether dietary supplementation with microalgae extracts from PT containing 4.4 mg/d of fucoxanthin affects changes in body composition or health markers in overweight women during an exercise and diet intervention. Materials and Methods: A total of 37 females (28.6 ± 7.9 years, 80.2 ± 14.9 kg, 29.6 ± 3.8 kg/m², 41.4 ± 4.2% fat) fasted for 12 h, donated a fasting blood sample, completed health and mood state inventories, and undertook body composition, health, and exercise assessments. In a counterbalanced, randomized, and double-blind manner, participants ingested a placebo (PL), or microalgae extract of *Phaeodactylum tricornutum* standardized to 4.4 mg of fucoxanthin (FX) for 12 weeks while participating in a supervised exercise program that included resistance-training and walking (3 days/week) with encouragement to accumulate 10,000 steps/day on remaining days of the week. The diet intervention involved reducing energy intake by about −300 kcal/d (i.e., ≈1400–1600 kcals/d, 55% carbohydrate, 30% fat, 15% protein) to promote a −500 kcal/d energy deficit with exercise. Follow-up testing was performed at 6 and 12 weeks. A general linear model (GLM) with repeated measures statistical analysis was used to analyze group responses and changes from baseline with 95% confidence intervals. Results: Dietary supplementation with microalgae extract from PT containing fucoxanthin for 12 weeks did not promote additional weight loss or fat loss in overweight but otherwise healthy females initiating an exercise and diet intervention designed to promote modest weight loss. However, fucoxanthin supplementation preserved bone mass, increased bone density, and saw greater improvements in walking steps/day, resting heart rate, aerobic capacity, blood lipid profiles, adherence to diet goals, functional activity tolerance, and measures of quality of life. Consequently, there appears to be some benefit to supplementing microalgae extract from PT containing fucoxanthin during a diet and exercise program. Registered clinical trial #NCT04761406.

## 1. Introduction

Obesity has become a pandemic, impacting over 200 million men and 300 million women worldwide, constituting 10% of the adult population [1,2,3,4]. Recognized as the leading preventable cause of mortality globally, obesity is associated with numerous medical comorbidities. Overweight and obesity prevalence varies within and between countries, with a higher prevalence in women than men overall [5,6]. The postmenopausal period exacerbates the challenges linked to overweight, obesity, and their health consequences in women [7]. Postmenopausal physiological changes, such as fat mass redistribution and muscle and bone mass loss, contribute to decreased physical activity, rendering women more susceptible to falls, hip fractures, insulin resistance, and abnormal glucose metabolism [8,9]. These conditions result in increased healthcare expenditures. Identifying preventive strategies that facilitate fat loss while preserving resting energy expenditure, lean tissue mass, bone mass, and improving strength and exercise capacity in premenopausal women holds substantial public health implications in helping manage these issues after menopause and as women age. Traditionally, weight-loss interventions have emphasized dietary energy restriction and increasing energy from low- to moderate-intensity exercise. Studies emphasize the role of energy restriction in promoting initial weight loss, with exercise playing a crucial role in weight loss maintenance and improved body composition parameters [10,11]. Combining diet and exercise is a fundamental aspect of weight management interventions. Beyond weight and fat mass loss, comprehensive weight management interventions should prioritize overall health improvement by reducing cardiovascular risk, enhancing exercise and functional capacity, and preventing musculoskeletal injuries—the most common adverse side effects of exercise in overweight populations. This comprehensive approach is essential for promoting long-term benefits and adherence to a healthy lifestyle.

Interestingly, behavioral-based interventions are more effective in combination with prescribed drugs or dietary supplements at reducing body fat storage and energy expenditure [12,13]. While various dietary supplements and pharmacological products have been explored to enhance adherence to weight management interventions and expedite weight loss, the efficacy of many of these strategies is unclear. Some of these approaches include fiber complexes, *Garcinia cambogia*, green tea, *Irvingia Gabonensis*, L-carnitine, herbal extracts, and marine product-based supplements rich in bioactive compounds. While some of these nutrients have been reported to promote weight loss, most have been evaluated without considering their combination or comparison with exercise and/or diet interventions. To address this gap, several studies indicate that combining exercise, diet, and nutritional strategies to promote weight loss has synergistic benefits for body composition and cardiorespiratory fitness in overweight and obese populations [14,15].

Prior studies indicate that marine algae and fucoxanthinol possess anti-obesity [16,17,18,19,20,21], lipid-lowering [17,19,22,23,24,25,26,27], and glucose management-enhancing properties through the regulation of inflammatory pathways [18,19,28,29]. For example, fucoxanthin was found to stimulate mitochondrial uncoupling protein 1 (UCP1) and β3-adrenergic receptor (Adrb3), responsible for lipolysis and thermogenesis [23,29]. Adaptive thermogenesis plays a critical role in energy expenditure by promoting heat production, weight maintenance, and promoting weight loss [30,31]. Additionally, increasing Adrb3 sensitivity to sympathetic nerve stimulation has been suggested to promote fat oxidation in white adipose tissue [30]. Because of the antioxidant and anti-inflammatory capacities of fucoxanthin and other compounds found in microalgae like *Phaeodactylum tricornutum* (e.g., polyunsaturated omega-3 fatty acids (n-3 PUFA), eicosapentaenoic acid (EPA), docosahexaenoic acid (DHA), and phycoprostans), microalgae supplementation may have additional benefits for cardiorespiratory fitness, lipid and glucose metabolism, and joint health, which may benefit individuals initiating an exercise and weight loss intervention [32].

While these findings are interesting, we are only aware of two studies to have examined the effects of dietary supplementation with fucoxanthin as a primary ingredient on weight management in overweight adults [33,34]. Abidov and coworkers [33,34] reported that supplementation with 1.8–8 mg/d of fucoxanthin increased resting energy expenditure and promoted greater weight loss in pre-menopausal overweight and obese individuals. However, since body composition was not assessed, it is unclear whether this was due to fluid, fat, and/or muscle loss. Additionally, Hitoe and associates [34] reported that fucoxanthin supplementation (1 and 3 mg/d for 4 weeks) significantly reduced body weight and scanned regional, visceral, and subcutaneous fat. However, neither of these studies involved exercise or diet intervention. Therefore, we hypothesized that dietary supplementation with a microalgae extract containing fucoxanthin during an exercise and diet intervention would promote greater weight loss, fat loss, and improved health markers. Hence, the primary objective of this proof-of-concept study was to investigate whether dietary supplementation with a microalgae-based ingredient containing fucoxanthin enhances the benefits in healthy overweight women engaged in exercise and dietary interventions on body composition parameters. Secondary objectives were to determine whether supplementation with fucoxanthin affects exercise and diet-induced changes in aerobic capacity, muscular strength and endurance, inflammation and oxidative stress biomarkers, hemodynamic status, and appetite-regulating hormones. The following describes the methods and results of this study and discusses the implications of the observed findings.

## 2. Methods

### 2.1. Research Design

**Figure 1:**
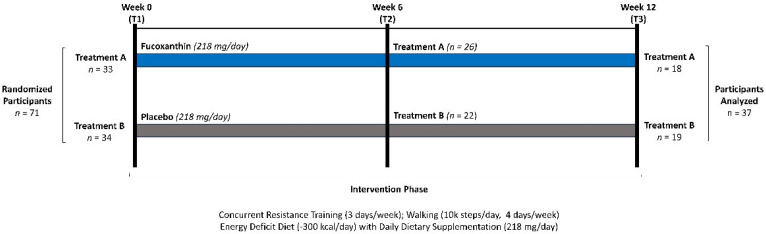
Overview of experiment study design.

This study was carried out as a randomized, counterbalanced, double-blind, placebo-controlled, parallel-group study (Figure 1). Participants followed a 12-week weight loss and supervised exercise program (3 days/week). The independent variable was dietary supplementation. The primary outcomes were changes in body composition and bone density. Secondary outcomes were resting energy expenditure (REE), peak aerobic capacity (VO_2peak_), bench press (BP) and leg press (LP) maximal strength (1RM) and endurance, hemodynamic markers, markers of oxidative stress, and inflammation and appetite-regulating hormones. All testing was conducted at the Exercise and Sport Nutrition Laboratory (ESNL) housed in the Human Clinical Research Facility (HCRF) at Texas A&M University. Exercise training sessions were conducted and supervised in the HCRF training facility by trained personnel. All phlebotomy procedures and blood processing procedures were conducted in biological safety level-2 laboratories and the fluxomics and metabolomics unit of the HCRF.

### 2.2. Study Participants

Sedentary healthy women aged 18–50 were recruited to take part in this randomized, double-blind, counterbalanced, placebo-controlled 12-week training and supplementation clinical trial. Study approval came from the Internal Review Board of Texas A&M University (IRB #2020-1443F). Participant recruitment from Texas A&M University and the surrounding area was achieved using mass email advertisements, flyers, and social media postings. Interested individuals were pre-screened before being invited to complete familiarization testing wherein study procedures were reviewed, informed consent documents were signed, medical histories were gathered, and physical exams were performed by a research assistant to evaluate eligibility. Inclusion criteria were pre-menopausal females (18 to 50 years), body mass index (BMI) of 25–35 kg/m^2^ and/or a body fat percentage greater than 30%, free living, in good health, and were willing to provide voluntary, written, informed consent to participate. Interested individuals were not allowed to participate if they were pregnant, not willing to provide informed consent, or diagnosed with a condition that may limit participation in a weight training program. Participants who met eligibility criteria signed informed consent statements in compliance with the Declaration of Helsinki and the Human Research Protection Program at Texas A&M University. This study was registered with clinicaltrials.gov (NCT04761406).

**Figure 2:**
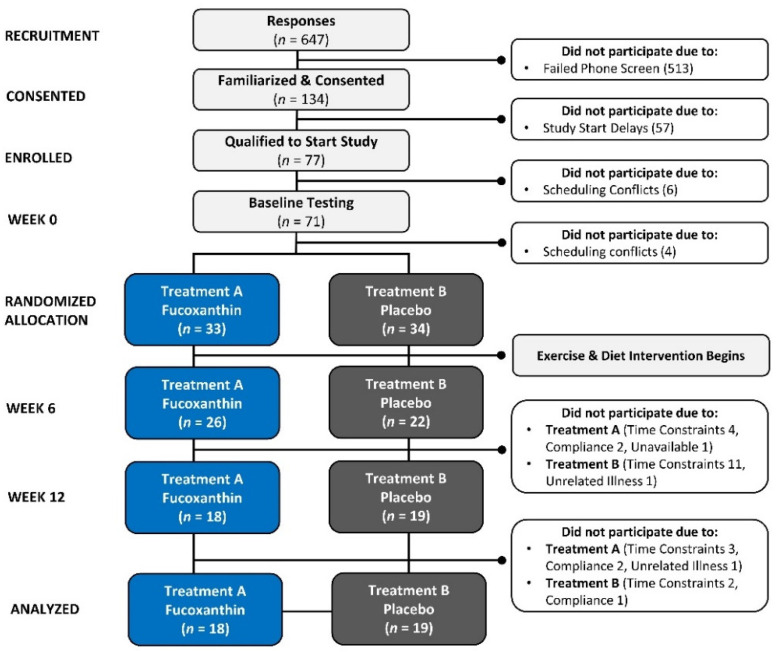
Consolidated Standards of Reporting Trials (CONSORT) flow chart for recruitment, randomization, allocation, completion, and analysis of the treatment groups. Unblinding groups revealed that Treatment A was Fucoxanthin and Treatment B was Placebo.

Figure 2 depicts a Consolidated Standards of Reporting Trials (CONSORT) diagram. Altogether, 647 people replied to advertisements for the study and were evaluated for eligibility. Of these, 134 met screening criteria and were invited to familiarization sessions. Owing to scheduling conflicts and start date delays, 77 women were familiarized and provided consent to take part in the study. Of these, four participants had scheduling conflicts and two decided not to participate. Thus, 71 individuals were randomly assigned to treatments. A total of 33 females were allocated to treatment A (FX) and 34 women were allocated to treatment B (PL). With treatment A, 26 participants completed 6 weeks of the study and 18 completed 12 weeks of the study. In treatment B, 22 participants completed 6 weeks and 19 completed the 12-week study. Reasons for not continuing to participate in the study were due to scheduling/time constraints (*n* = 20), non-compliance (*n* = 5), availability (*n* = 1), and unrelated illness (*n* = 2). A total of 13 participants withdrew from treatment A and 15 participants withdrew from treatment B.

### 2.3. Testing Protocol

**Figure 3:**
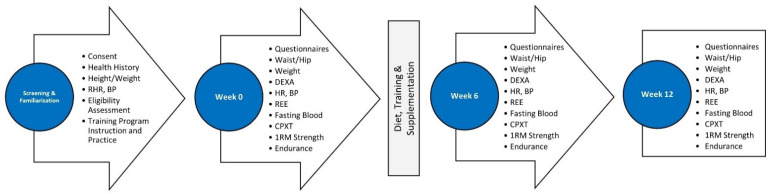
Testing sequence and timeline. RHR = resting heart rate, BP = blood pressure, DEXA = dual -energy X-ray absorptiometry, REE = resting heart rate, CPXT = cardiopulmonary exercise test, 1RM = one repetition maximum.

Figure 3 outlines the order of testing conducted at each session. Participants attended four sessions including a familiarization and three experimental testing sessions at 0, 6, and 12 weeks. After the familiarization session, those eligible and who consented to take part were scheduled for a baseline testing, asked to document food and fluid intake for four days, refrain from vigorous exercise (48 h), and fast (12 h) prior to reporting to testing sessions. For baseline testing, resting measures were obtained consisting of height, weight, waist and hip circumferences, resting heart rate (RHR), resting blood pressure, REE, and dual-energy X-ray absorptiometry (DEXA)-determined body composition. Participants then completed an SF36 Quality of Life (QOL) inventory [35] and side effects questionnaires. A venous blood sample (≈20 mL) using standard venipuncture techniques was then taken. Participants then performed an incremental, symptom-limited, maximal cardiopulmonary exercise test (GPXT) on a treadmill to determine VO_2peak_. Participants then performed BP and LP one-repetition maximum (1RM) strength and muscular endurance tests at 70% 1RM. Participants repeated this testing at 6 and 12 weeks.

### 2.4. Familiarization

Once individuals responded to advertisements, they were contacted with a preliminary screening questionnaire to determine if they were eligible to participate in a familiarization session. Eligible individuals were invited to the ESNL for the initial consultation, which consisted of reviewing study procedures, signing informed consent documents, completing medical histories, and undergoing a physical exam involving ascertaining height, weight, resting heart rate, and blood pressure. If BMI was not between 25 and 35 kg/m^2^, body fat percentage was assessed via DEXA to assess if they were greater than 30% body fat to meet inclusion criterion. After resting measures were taken, instructions on logging and submitting dietary logs through MyFitnessPal were given. Once participants were recruited, they were randomly allocated to the supplementation groups.

### 2.5. Randomization

Participants were assigned to ingest either one soft gel or two powder-encased capsules daily of microalgae extract containing FX for 12 weeks or matching placebo (PL) ingredients. Treatments were counterbalanced based on BMI, body fat percentage, and age and provided numerically ordered supplement packages that were randomized for double-blind administration. Both groups followed the same concurrent exercise protocol. Additionally, all participants were instructed to maintain a 300 kcal/day deficit diet based on REE measures taken at baseline and six weeks.

### 2.6. Training Intervention

Participants followed a progressive resistance and cardiovascular training program three days/week for 12 weeks. Sessions were supervised by trained group fitness instructors and/or lab personnel. Training logs were provided to track performance and progress. Resistance training consisted of 11 upper and lower extremity exercises emphasizing all major muscle groups using free weights, machines, or calisthenics, and progressively increasing training volume from weeks 1 to 12. Each exercise progressed in a 4 week periodized manner that varied from 2 to 3 sets of 10 to 8 repetitions with 2 min rests between each set and exercise. Increases in resistance were encouraged bi-weekly, if able, and spotters were available if needed. The cardiovascular training consisted of 20 min at 60–80% heart rate reserve (HRR) using a treadmill, stationary bike, or outside running/walking. Heart rate reserve was calculated from CPXT maximal heart rate and baseline resting heart rate (HR_max_ and HR_rest_, respectively) using the following formula: ((HR_max_ − HR_rest_) × 0.6 or 0.8) + HR_rest_. Participants were supplied with a H10 heart rate monitor and watch (Polar Electro Inc., Bethpage, NY, USA) to monitor HR for compliance and safety. Distance travelled, total exercise time, and average HR were recorded on training days. Participants adjusted cardiovascular workloads to maintain the prescribed exercise range. Exercise program compliance was set at a minimum of 70% (25/36 sessions) [36]. If a participant did not reach the 70% adherence rate, they were dropped from the study. On non-training days, participants were asked to walk 10,000 steps/day which were tracked mostly from Apple or Samsung smart watches and their accompanying smartphone applications or through a provided clip-on pedometer (BATAUU, Shenzhen, China). Step compliance was monitored and recorded on non-supervised training days.

### 2.7. Diet Intervention

Participants were given a 300 kcal/day deficit diet based on resting caloric expenditure determined during REE assessment at baseline. Calories were subtracted from REE with approximately 200 more calories being expended daily through walking and training, which equated to approximately a 500 kcal/day deficit total. Participants were asked to consume a diet consisting of approximately 55% carbohydrate, 15% protein, and 30% fat daily. Registered dietitian prepared meal plan examples and food and beverage exchange lists, consistent with the United States Department of Agriculture and American Heart Association (AHA) dietary guidelines, were given as resources to the participants so they could add variety to the diets. Participants recorded dietary intake using handwritten food logs or MyFitnessPal (MyFitnessPal, Inc., Baltimore, MD, USA).

### 2.8. Supplementation Protocol

Participants were randomized to ingest daily either placebo or 4.4 mg FX, as one soft gel containing 220 mg microalgae extract from *Phaeodactylum tricornutum* standardized to 2% (4.4 mg) of FX (PhaeoSol™, Microphyt, Baillargues, France) blended with 220 mg of medium chain triglycerides (MCT) oil, or encased capsules containing 275 mg of PT powder standardized 0.8% of FX for 12 weeks. The matching PL consisted of soft gel capsules containing 440 mg of sunflower oil or 275 mg of maltodextrin powder in encased capsules. The dosages used followed United States Food and Drug Administration approved dosages and were consistent with other studies conducted in humans. Placebos were color-matched refined sunflower oil and maltodextrin polysaccharide with food additive. Placebos were manufactured to appear identical and taste like the experimental supplement. The product manufacturers provided a certificate of analysis verifying dosage and absence of contaminants in the supplements. Supplementation began on the seminal day of training after baseline testing and were ingested daily at lunch with eight ounces of water. The supplements were soft gel and powdered form with half of the cohort ingesting soft gel capsules of the PL or FX and the other half ingesting encapsulated powder version. Both forms of supplementation were distributed in blister packets and stored at 4 °C. Supplementation compliance checks were conducted at each testing session and periodic check-ins. Compliance was also emphasized through frequent emails and verbal communication to participants. Participants ingested their given supplement at home apart from study days, where they consumed it on-site.

## 3. Procedures

### 3.1. Diet Assessment

Participants recorded dietary intake using MyFitnessPal (MyFitnessPal, Inc., Baltimore, MD, USA) [37]. Four-day (3 weekdays and 1 weekend day) diet records were assessed by research assistants using version 11.14.9 of the Food Processor Nutrition Analysis Software (ESHA Nutrition Research, Salem, OR, USA) [38,39].

### 3.2. Anthropometrics and Hemodynamics

Height (cm) and weight (kg) were taken with a digital Health-O-Meter, self-calibrating (±0.02 kg), Professional 500KL (Pelstar LLC, Alsip, IL, USA) scale. Waist and hip circumferences were measured using Gulick tape measures following standard procedures [40]. Resting HR and BP were measured in a supine position, following 2–6 min of rest, with a digital blood pressure cuff (Connex^®^ ProBP™ 3400; Welch Allyn, Tilburg, The Netherlands).

### 3.3. Body Composition

Body composition, excluding cranium (i.e., fat mass, lean tissue mass, BMC, BMD, bone mineral area (BMA), and gynoidal, appendicular, and visceral adipose tissue volume and area) were measured with a calibrated Hologic Discovery W DEXA (Hologic Inc., Waltham, MA, USA) using APEX Systems Software (version 4.0.2., APEX Corporation Software, Pittsburg, PA, USA) [41,42]. Test–retest reliability from our lab show C_V_ ranges of 0.31–0.45% for BMC, total mass, and FFM with mean intraclass correlation of 0.98 [43].

### 3.4. Resting Energy Expenditure Assessment

Resting energy expenditure was assessed using standard procedures with a ParvoMedics TrueOne 2400 Metabolic Measurement System (ParvoMedics Inc., Sandy, UT, USA). Metabolic cart calibration followed standard procedures, with a three-liter series 5530 syringe (Hans Rudolph Inc., Kansas City, MO, USA). Individuals were supine with their knees and hips bent at 90°, elevated on a cushioned cube, and asked to stay awake for 20–30 min and relax. After 10 min and observation that values were stabilized within a 5% variance, five timepoints were averaged to calculate REE values [44,45]. Utilization of glucose and fatty acids were calculated via the non-protein respiratory quotient (RQ) [46,47]. A C_V_ of 5.3% with an interclass correlation of 0.92 has been reported in female athletes [48], while a C_V_ of ±2% is reported by the manufacturer for apparently healthy individuals.

### 3.5. Exercise Assessment

Peak oxygen uptake was determined on a motorized treadmill (TrackMaster 425, Newton, KS, USA) using the Bruce protocol [35] until volitional fatigue at perceived maximum. Expired ventilation and oxygen content were measured using a metabolic measurement care (TrueOne 2400, ParvoMedics Inc., Sandy, UT, USA). The pneumotach was calibrated with a series 5530 calibration syringe (Hans Rudolph Inc., Kansas City, MO, USA) while oxygen and carbon dioxide sensors were calibrated to certified medical grade gas following standard procedures. Heart rate and rhythm were monitored using a Cardio-Card version 7.2 electrocardiograph (NasifF Associates, Brewerton, NY, USA) while perceptions of fatigue were monitored using the Borg 6–20 Rating of Perceived Exertion (RPE) scale. Upon volitational fatigue, participants had a 10 min cooldown. Participants then performed a BP and LP one-repetition maximum (1RM) strength test on standard bench and hip sled/leg presses (Nebula Fitness, Versailles, OH, USA). Strength tests were performed using standard protocols, with appropriate rest periods between sets [49]. After a 3 min rest, participants performed a muscle endurance repetition test to failure at 70% 1RM.

### 3.6. Blood Collection and Analysis

Fasting whole blood sample (≈20 mL) collection utilized standard phlebotomy procedures [50] by certified phlebotomists, into three BD (Becton, Dickinson and Company, Franklin Lakes, NJ, USA) Vacutainer serum separation tubes (SSTs) and one BD Vacutainer ethylenediaminetetraacetic acid (EDTA) tube, 7.5 and 3.5 mL, respectively. The SSTs were stored at room temperature for about 15 min and then centrifuged at 3000× *g* for ten minutes using a refrigerated (4 °C) centrifuge (MegaFuge 40R, Thermo Scientific Heracus, West Palm Beach, FL, USA). Serum from the SSTs were aliquoted into polypropylene Eppendorf microcentrifuge tubes (Eppendorf, Enfield, CT, USA) and stored at −80 °C for further analysis. The remaining SST and EDTA tubes were sent to the Clinical Pathology Laboratory (Bryan, TX, USA) for comprehensive blood count with differentiation and chemistry panels. Serum leptin and insulin were measured via commercially available (Alpco Diagnostics, Salem, NH, USA) enzyme-linked immunoassay (ELISA) kits and a BioTek Epoch 2 plate reader with BioTek Gen 5 software (BioTek Instruments, Winooski, VT, USA), according to company instructions. Intra- and inter-assay C_V_’s were 3.2–10.3% and 6.7–16.6% at 5.8–140.9 ng/mL for insulin and 2.2–4.0% and 5.1–8.5% at 2.5–54.5 ng/mL for leptin. Measures of insulin sensitivity (i.e., glucose to insulin ratio (GIR), homeostatic model assessment for insulin resistance (HOMA_IR_), quantitative insulin sensitivity check index (QUICKI)) were calculated using standard equations [51]. Serum cytokines, i.e., interleukin (IL-) 1β, 2, 4, 5, 6, 8, and 10, interferon-γ (IFN-γ), tumor necrosis factor-α (TNF-α), and granulocyte-macrophage colony-stimulating factor (GMC-SF) were measured with a commercially available Cytokine Human Magnetic 10-plex Panel, on a Luminex 200 Instrument System and a Milliplex Analyzer (ThermoFisher Scientific, Vienna, Austria) using xPONENT^TM^ software (version 3.1), following company instructions. Inter- and Intra-assay calculations from previous experiments from our lab observed C_V_’s of 2.2–17.5% and 3.3–9.8%, respectively.

### 3.7. Quality of Life

Subjective perceptions on quality of life (QOL) were assessed via the Short Form Health Survey version 2 (SF-36v2) [52]. This survey contains generic quality of life questions covering various domains of psychological and physical health: vitality, social functioning, role emotional health, and mental health. The SF-36v2 inventory has shown a test–retest reliability of r = 0.81–0.95 for all domains [53,54].

### 3.8. Side Effects

Side effect frequency and severity (i.e., dizziness, headache, tachycardia, palpitations, shortness of breath, nervousness, blurred vision, and other) were assessed, respectively, using separate Likert-scales where 0 = none; 1 = 1–2 per week or minimal; 2 = 3–4 per week or slight; 3 = 5–6 per week or moderate; 4 = 7–8 per week or severe; and 5 = ≥9 per week or very severe. From our lab, the test–retest reliability of this assessment showed a C_V_ of 1.2–2.6% with single-item survey intraclass correlations between 0.6 and 0.88 [55,56,57].

### 3.9. Statistical Analysis

The IBM^®^ (IBM Corp., Armonk, NY, USA) Version 29 SPSS^®^ statistical software was used for data analysis. Sample size selection was based on our prior related work in this area [58,59] assuming a 5% improvement with an 80% power in primary outcome weight and body composition variables. Multivariate and univariate general linear model (GLM) with repeated measures analysis for time and groups was utilized to examine data. Mauchly’s test assessed sphericity, while normality was looked at with skewness and kurtosis statistics. Time (T) and group × time (GxT) interaction effects were assessed using the Wilks’ Lambda and Greenhouse–Geisser univariate correction tests. The probability of type I error (*p*-level) was set at 0.05 or less. When *p*-values ranged between 0.05 and 0.10, they were noted as statistical tendencies. Pairwise differences with Fisher’s least significant difference (LSD) confidence interval (CI) adjustment were also assessed. Assessment of mean changes with 95% confidence intervals (CI) was used to evaluate the clinical significance of findings. Means and 95% CIs were considered statistically and clinically significant if they were completely above or below baseline [60]. Data in tables are displayed as means with standard deviations (SD). Data in figures are presented as mean changes from baseline with 95% CIs (LL, UL). Partial Eta squared (η_p_^2^) statistics were used to assess effect sizes (small 0.10, medium 0.06, large 0.14) [61]. Pearson’s Chi-Squared analysis was used to assess changes in the frequency distribution of side effects and QOL questionnaire answers from baseline. Missing data (<0.6%) were replaced with series means modified by group, or most commonly reported number for frequency data.

## 4. Results

### 4.1. Participant Demographics

Table S1 shows demographic data. Participants were 28.6 ± 7.9 years, 164.0 ± 7.8 cm, weighed 80.2 ± 14.9 kg, had a BMI of 29.6 ± 3.8 kg/m², and 41.4 ± 4.2% body fat. No significant overall (*p* = 0.736) or univariate differences between the groups were observed in age, height, weight, waist and hip circumferences, resting heart rate, or systolic and diastolic blood pressure.

### 4.2. Energy and Macronutrient Intake

**Figure 4:**
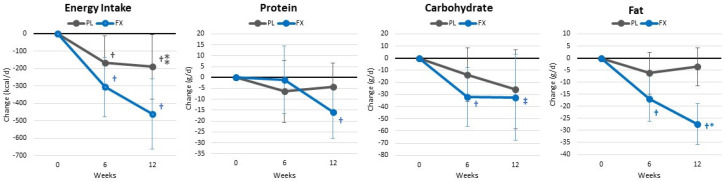
Changes in energy and macronutrient intake. Data are means and ± 95% confidence intervals. PL = placebo, FX = fucoxanthin-containing supplement, † = *p* < 0.05 (‡ = *p* > 0.05 to <0.10) from baseline. ⁎ = *p* < 0.05 (⁑ = *p* > 0.05 to *p* < 0.10) difference between groups.

Table S2 shows energy and macronutrient results. Overall, GLM multivariate analysis revealed a significant time (*p* < 0.001, η_p_^2^ = 0.259) and group × time (*p* = 0.035, η_p_^2^ = 0.128) interaction effect. Univariate analysis showed that dieting significantly decreased energy intake, protein, carbohydrate, and fat intake. Reductions in total energy intake (*p* = 0.076, η_p_^2^ = 0.084, moderate effect) and fat (*p* = 0.001, η_p_^2^ = 0.198, large effect) intake were greater in the FX group. These differences were also observed in mean changes from baseline (±95% CIs) (see Figure 4).

### 4.3. Training Volume

Table S3 presents training volume data from the resistance and endurance training programs. Multivariate GLM analysis showed significant time (*p* < 0.01, η_p_^2^ = 0.750, large effect) but no group × time (*p* = 0.286, η_p_^2^ = 0.036, small effect) interaction effects between the groups. Univariate analysis showed similar findings. A GLM analysis of exercise heart rate during supervised walking and the number or steps/day showed no significant time (*p* < 0.313, η_p_^2^ = 0.034, small effect) effects while group × time effects (*p* = 0.086, η_p_^2^ = 0.057, medium effect) tended to interact. A post hoc analysis showed that the number of steps/week increased in the FX group from baseline while being unchanged with PL treatment.

### 4.4. Body Composition and Anthropometric Measures

**Figure 5:**
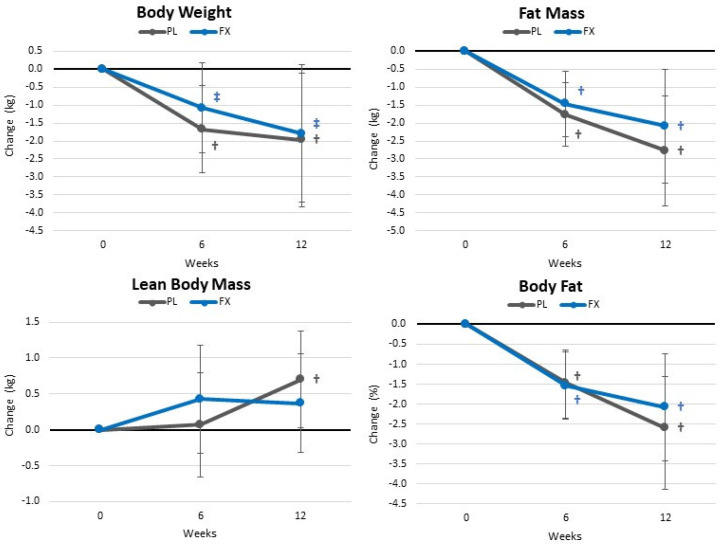
Changes in body weight and composition. Data are means and ± 95% confidence intervals. PL = placebo, FX = fucoxanthin containing supplement, † = *p* < 0.05 (‡ = *p* > 0.05 to <0.10) from baseline.

Table S4 shows body composition and anthropometric measurement results. Overall, GLM analysis showed significant time (*p* < 0.001, η_p_^2^ = 0.275, large effect) but no group × time effects (*p* = 0.915, η_p_^2^ = 0.054, small effect). When expressed as changes from baseline (±95% CIs) (Figure 5), both groups observed weight and fat loss while gaining FFM. However, no statistically significant group differences were seen.

**Figure 6:**
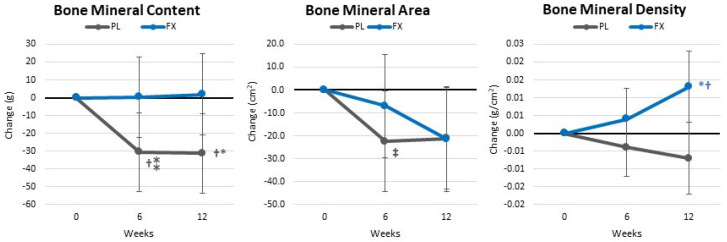
Changes in body weight and composition. Data are means and ± 95% confidence intervals. PL = placebo, FX = fucoxanthin containing supplement, † = *p* < 0.05 (‡ = *p* > 0.05 to <0.10) from baseline. ⁎ = *p* < 0.05 (⁑ = *p* > 0.05 to <0.10) difference between groups.

Table S5 shows bone-related variables. GLM analysis found no significant time effects (*p* = 0.197, η_p_^2^ = 0.061, medium effect). However, a significant interaction effect (*p* = 0.023, η_p_^2^ = 0.101, medium effect) was observed among BMC, BMD, and BMA. Pairwise comparison found a significant decrease in BMC, BMD, and BMA in the placebo group. However, those taking FX maintained BMC while experiencing a significantly increase BMD (interaction *p* = 0.010, η_p_^2^ = 0.126, medium effect). An analysis of the mean changes from baseline (±95% CIs) indicated that the differences in BMC (−33.1 [−65.1, −1.1] gm, *p* = 0.043) and BMD (0.020 [0.006, 0.034] gm/cm^2^, *p* = 0.006) after 12 weeks were significantly different between the groups, with those supplementing the diet with FX maintaining BMC and increasing BMD compared with those ingesting a PL during the exercise and weight loss intervention (see Figure 6).

### 4.5. Resting Energy Expenditure and Metabolism

**Figure 7:**
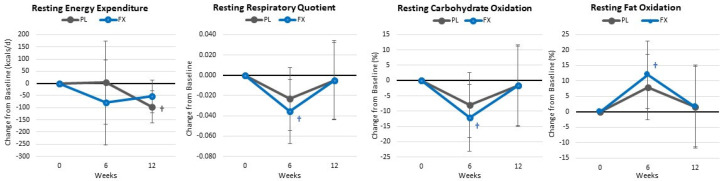
Changes in resting energy expenditure, respiratory quotient, and substrate oxidation. Data are means and ±95% confidence intervals. PL = placebo, FX = fucoxanthin containing supplement, † = *p* < 0.05 from baseline.

Table S6 presents resting REE and substrate oxidation data. Overall, GLM analysis showed a significant time (*p* < 0.09, η_p_^2^ = 0.076, moderate effect) but no group × time (*p* = 0.702, η_p_^2^ = 0.027, small effect). Likewise, no significant group x time interaction effects were seen in absolute values or when expressed as mean changes from baseline (±95% CIs). However, as seen in Figure 7, RQ and carbohydrate oxidation decreased while fat oxidation increased (12.1 [10.9, 23.0] %, *p* = 0.031) after 6 weeks of supplementation in the FX group without a significant decline in REE. Conversely, participants in the PL group observed a clinically significant decline in REE with no change from baseline in RQ or carbohydrate and fat oxidation.

### 4.6. Exercise and Functional Capacity Assessment

**Figure 8:**
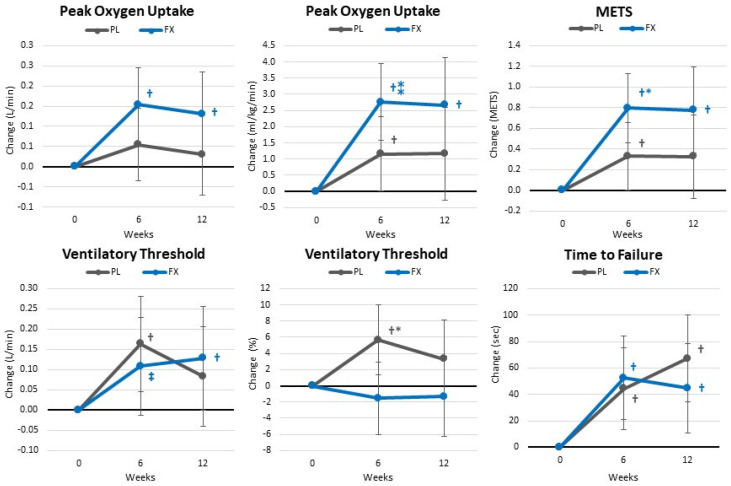
Changes in peak oxygen uptake and ventilatory threshold. Data are means and ± 95% confidence intervals. PL = placebo, FX = fucoxanthin containing supplement, † = *p* < 0.05 (‡ = *p* > 0.05 to <0.10) from baseline. ⁎ = *p* < 0.05 (⁑ = *p* > 0.05 to <0.10) difference between groups.

Table S7 shows the results from the cardiopulmonary exercise test. Overall, GLM analysis showed a significant time (*p* < 0.001, η_p_^2^ = 0.260, large effect) and group × time (*p* = 0.032, η_p_^2^ = 0.154, large effect). Pairwise comparison analysis showed that training increased aerobic capacity in both groups with greater gains observed in the FX group, particularly in VO_2peak_ and ventilatory anaerobic threshold after 6 weeks of supplementation (see Figure 8).

**Figure 9:**
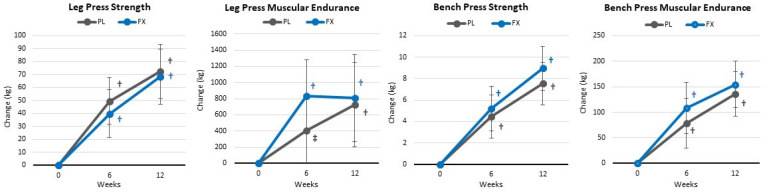
Changes in muscular strength and endurance. Data are means and ± 95% confidence intervals. PL = placebo, FX = fucoxanthin containing supplement, † = *p* < 0.05 (‡ = *p* > 0.05 to<0.10) from baseline.

Table S8 presents isotonic strength and endurance results. Overall, GLM analysis showed a significant time (*p* < 0.001, η_p_^2^ = 0.575, large effect) with no significant group × time effects (*p* = 0.666, η_p_^2^ = 0.041, small effect). Similarly, univariate analysis showed significant time effects in muscular strength and endurance assessments with no significant interactions observed between the groups. Similar findings were seen when analyzing mean changes from baseline (±95% CIs) (see Figure 9).

### 4.7. Blood Sample Analysis

Table S9 presents whole blood cell blood count results. Overall, GLM analysis showed a non-significant time (*p* = 0.850, η_p_^2^ = 0.149, large effect) and group × time (*p* = 0.177, η_p_^2^ = 0.240, large effect). Univariate analysis showed no significant time or group × time effects in whole blood markers except for eosinophils, which decreased in the FX group (*p* = 0.042, η_p_^2^ = 0.08, medium effect). A pairwise comparison showed lymphocytes, monocytes, and eosinophils as significantly higher in the FX group. However, these values were well within normal ranges for active individuals.

Table S10 presents serum metabolic panels. Overall, GLM analysis showed a non-significant time (*p* = 0.159, η_p_^2^ = 0.290, large effect) and group × time (*p* = 0.832, η_p_^2^ = 0.191, large effect). Univariate analysis showed no significant time or group × time effects. Though, a pairwise comparison showed significant time effects in several whole blood markers with creatinine, globulin, and the albumin to globulin ratio (A:G ratio) increasing in the PL group. At 6 weeks, calcium albumin, and the A:G ratio were higher with PL.

**Figure 10:**
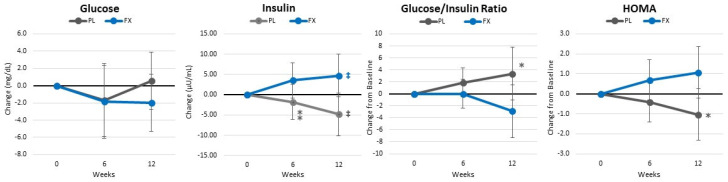
Changes in markers of glucose homeostasis and insulin sensitivity. Data are means and ± 95% confidence intervals. PL = placebo, FX = fucoxanthin containing supplement, ‡ = *p* > 0.05 to <0.10 from baseline, * = *p* > 0.05 (⁑ = *p* > 0.05 to <0.10) difference between groups.

Table S11 presents markers of glucose homeostasis and insulin sensitivity. Overall, GLM analysis showed a non-significant time (*p* = 0.434, η_p_^2^ = 0.076, medium effect) or group × time effects (*p* = 0.093, η_p_^2^ = 0.116, medium effect). However, univariate analysis showed significant group × time effects in insulin, the GIR, HOMA_IR_, and QUICKI. These changes are also seen when analyzing changes from baseline (±95% CIs) (Figure 10).

**Figure 11:**
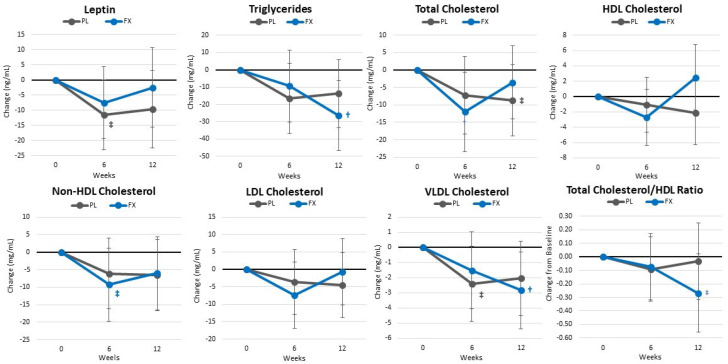
Changes in blood lipids. Data are means and ±95% confidence intervals. PL = placebo, FX = fucoxanthin containing supplement, † = *p* < 0.05 (‡ = *p* > 0.05 to <0.10) from baseline.

Table S12 presents lipid-related markers. Overall, GLM analysis showed non-significant time (*p* = 0.178, η_p_^2^ = 0.146, large effect) or group × time effects (*p* = 0.518, η_p_^2^ = 0.107, medium effect). No significant interactions were observed from the univariate analyses. However, HDL levels tended to increase (7.72 [−1.083, 16.53] mg/dL, *p* = 0.084) and the ratio of LDL to HDL tended to be lower (−0.488 [−0.98, 0.08], *p* = 0.095) after 12 weeks of FX supplementation. Figure 11 shows mean changes from baseline in these markers. The appetite hormone leptin and VLDL tended to decrease to a greater degree after 6 weeks in the PL group. In the FX group, non-HDL cholesterol tended to decrease after 6 weeks while triglycerides, VLDL cholesterol, and the ratio of total cholesterol to HDL cholesterol significantly decreased after 12 weeks. However, no significant differences were seen in changes from baseline between groups.

**Figure 12:**
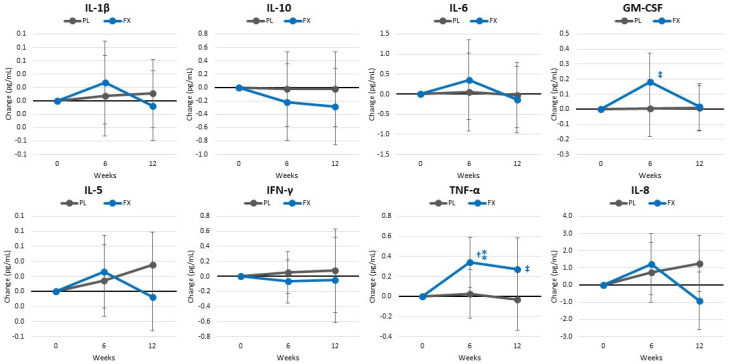
Changes in cytokines and inflammatory markers. Data are means and ±95% confidence intervals. PL = placebo, FX = fucoxanthin containing supplement, † = *p* < 0.05 (‡ = *p* > 0.05 to <0.10) from baseline. ⁑ = *p* > 0.05 to <0.10) difference between groups.

Table S13 presents cytokine and inflammatory markers. Overall, GLM analysis showed that time effects tended to change (*p* = 0.085, η_p_^2^ = 0.167, large effect) with no significant group × time effects (*p* = 0.137, η_p_^2^ = 0.154, large effect). Univariate analysis showed some evidence that IL-2, IL-6, and TNF-α increased to a greater degree with FX. Similar trends were observed when assessing mean changes from baseline (±95% CIs) (Figure 12).

### 4.8. Quality of Life

Table S14 presents the SF-36 Quality of Life data. No significant relationships were observed from the Chi-squared analysis between groups and participant responses to all questions. At baseline, participants in the FX group rated bending, kneeling, and stooping to be more limited (*p* = 0.047); however, this perception became non-significant after supplementation began. Similarly, participants in the FX group rated more difficulty walking several hundred yards (*p* = 0.063) than those in the PL group prior to the study starting with these differences becoming not significant after supplementation began. There was also some evidence that participants experienced differences in rating the amount of pain interfering in normal activities, the amount of time feeling full of life (*p* = 0.088), and becoming sick easier than others (*p* = 0.097), with perceptions more favorable with FX supplementation.

### 4.9. Resting Heart Rate and Blood Pressure

Table S15 shows resting hemodynamic results. Resting heart rates tended to decrease in both groups over time with no significant differences observed between groups. In this regard, resting heart rate significantly decreased from baseline after 6 weeks (−6.8 [−12.4, −1.3] beats/min, *p* = 0.017) and 12 weeks (−7.3 [−13.1, −1.5] beats/min, *p* = 0.015) and tended to be lower after 6 weeks (−6.3 [−14.0, 1.5] beats/min, *p* = 0.109) and 12 weeks (−6.3 [−14.9, 1.3] beats/min, *p* = 0.097) compared with PL values. No significant differences were seen in resting systolic or diastolic blood pressure during the study.

### 4.10. Side Effects

Table S3 presents side effect data. After 6 weeks of supplementation, participants in the FX group felt a greater number of infrequent heart palpitations (*n* = 4, 1–2 per week, *p* = 0.03) while the frequency of blurred vision decreased in frequency with FX. In terms of the severity of side effects, more participants in the FX group reported minimal severity of dizziness after 6 (*p* = 0.047) and 12 weeks of supplementation (*p* = 0.051) and minimal severity of heart palpitations (*p* = 0.094). No participant withdrew from the study due to a lack of tolerance to the supplement.

## 5. Discussion

Marine algae and fucoxanthinol have been reported to have anti-obesity [16,17,18,19,20,21,23,29], lipid-lowering [17,19,22,23,24,25,26,27], and glucose-management properties [18,19,28,29]. Theoretically, dietary supplementation with a microalgae extract from *Phaeodactylum tricornutum* containing fucoxanthin could promote greater fat loss and/or improvement in health outcomes during an exercise and weight loss intervention. This study examined whether the daily consumption of a microalgae extract from *Phaeodactylum tricornutum* standardized to provide 4.4 mg/d of fucoxanthin affected weight loss and/or markers of health in sedentary overweight women initiating an exercise and weight loss diet intervention. The results showed that while supplementing the diet with fucoxanthin tended to help participants adhere to the diet intervention, there was no additional benefit to weight loss or body composition (primary outcomes). However, fucoxanthin supplementation preserved bone mass and density to a greater degree and promoted greater improvements in walking steps/day, resting heart rate, aerobic capacity, blood lipid profiles, adherence to diet goals, functional activity tolerance, pain, and measures of quality of life. Consequently, there appears to be some benefit to the dietary supplementation of fucoxanthin while taking part in a diet and exercise program. The following further discusses the results in the context of the related literature, limitations, and needs for further research.

### 5.1. Primary Outcomes

Marine algae and seaweed containing fucoxanthin have been reported to possess anti-obesity properties [16,18,19,20,21,62,63] by increasing fat oxidation through the upregulation of UCP1 expression in white adipose tissue [23,64,65], increasing lipolytic enzyme activity [20,66,67], and suppressing obesity-related inflammation [18,68,69,70]. Theoretically, consuming a microalgae containing fucoxanthin during an exercise and weight loss program may increase thermogenesis and promote fat oxidation and white adipose tissue loss [71]. In support of this contention, Maeda and coworkers [23] reported that feeding rats lipids from seaweed containing fucoxanthin for 4 weeks increased UCP1 expression and promoted a reduction in abdominal white adipose tissue weight. Additionally, feeding an obese/diabetic mouse model (KK-A^γ^) fucoxanthin for 4 weeks promoted a significant reduction in abdominal white adipose tissue compared with controls. In another study, Kang and coworkers [66] reported that the dietary feeding of *Petalonia binghamiae* extract as a source of fucoxanthin increased β-oxidation and reduced lipogenesis in mice leading to less weight gain over time. Collectively, although these studies were conducted in mice and rats, they provide a theoretical rationale that the dietary supplementation of fucoxanthin may affect fat oxidation, accumulation, and/or weight loss.

While these findings are interesting, we only know of two studies that have examined the effects of dietary supplementation with fucoxanthin as a primary ingredient on weight management in overweight adults [33,34]. In the first study, Abidov et al. [33] assessed the effects of 16 weeks of dietary supplementation with 300 mg of pomegranate oil and 300 mg brown seaweed extract providing 1.6–8 mg of fucoxanthin on weight management in pre-menopause. The researchers reported that compared with a placebo, supplementation with the fucoxanthin promoted greater weight loss (−1.4 kg vs. −6.3 kg, *p* < 0.05), reductions in liver fat content in individuals with non-alcoholic fatty liver disease, and a dose-dependent increase in REE (up to 1915 ± 246 kJ/24 h with 8 mg/d). These findings suggest that supplementing the diet with as little as 4–8 mg/d of fucoxanthin may promote weight loss. However, since body composition was not measured, it is unclear whether the weight loss was due to fluid, fat, or lean tissue mass loss. In the second study, Hitoe et al. [34] reported that fucoxanthin supplementation for 4 weeks (1 and 3 mg/d) significantly reduced body weight (−0.7 to −1.3 kg), bioelectrical impedance-determined fat mass (−1.2% to −3.5%), computed tomography scanned trunk (−5.7% and −3.8%), visceral (−6.1% to −16.3%), and subcutaneous (−5.3% and 1.5%) fat, respectively, in moderately obese adults without diet or exercise intervention. However, energy intake and REE were not affected with 4 weeks of 1 and 3 mg/d of fucoxanthin supplementation. Also, it should be noted that the statistical analysis of the results was limited and not provided in detail in this report.

In the present study, 12 weeks of walking, resistance training, and the maintenance of a modest decrease in energy intake promoted significant weight loss (−1.88 [−0.6, −3.2] kg, *p* = 0.007), fat loss (−2.4 [−1.3, −3.5] kg, *p* < 0.001), and a reduction in body fat percent (−2.43 [−1.3, −3.5] %, *p* < 0.001) while increasing FFM (0.54 [0.06, 1.01] kg, *p* = 0.03) in both treatment groups. These findings are consistent with our prior research [10,36,72,73,74,75,76,77] and others [78,79,80,81,82,83] that showed that this type of exercise and dietary intervention promotes a reduction in energy intake and fat loss while maintaining or increasing muscle mass and/or REE. However, beyond a trend toward a greater reduction in energy and a significant reduction in fat intake, supplementation with fucoxanthin did not promote a significant increase in REE or greater weight and fat loss compared with those ingesting a placebo. Whether the significant reduction in energy and fat intake observed would result in significant differences in weight and/or fat loss over time remains to be determined. However, results from this study do not support contentions that the dietary supplementation of 4.4 mg/d of fucoxanthin augments weight and/or fat loss in women participating in an exercise and diet intervention.

With that said, there was evidence that participants supplementing their diet with fucoxanthin preserved BMC and increased BMD density (1.4%) while those consuming the placebo observed a significant reduction in BMC (−1.9%) while maintaining BMD. Although the etiology remains to be determined, prolonged caloric restriction has been reported to promote clinically significant bone loss compared with individuals following their normal diet [83,84]. For example, Villareal [84] reported that adherence to an energy-restricted diet decreased BMD and impaired bone turnover in younger adults (20–50 years). The bone loss was partially explained by changes in body weight, fat and muscle loss, 25-hydroxyvitamin D status, markers of bone turnover, differences in hormones (cortisol, leptin, adiponectin, insulin), and less physical activity [84]. Serra and coworkers [83] reported that BMD decreased (−1.2%) in women dieting with and without aerobic exercise training. However, the 10% increase in VO_2peak_ in those participating in a walking exercise program (3 × 45-min/week at about 70% VO_2peak_) attenuated loss in BMD after energy restriction suggesting that aerobic capacity may influence bone maintenance during and after weight loss. Resistance-exercise has been reported to attenuate bone loss during energy-restricted diets [85]. In the present study, those taking fucoxanthin not only maintained BMC and increased BMD but also experienced a greater improvement in peak aerobic capacity. There was also evidence of a higher fasting insulin in the fucoxanthin group without significantly increasing insulin resistance. Insulin has been reported to have an anabolic effect [86,87] and there are insulin receptors in pre-osteoblasts and osteoblasts suggesting that insulin plays a role in the differentiation of osteoblasts [88,89] and targets osteoblasts to help control glucose homeostasis and regulate osteocalcin activation and production thereby enhancing bone resorption by osteoclasts [90,91]. There is also evidence that osteoclast-like cells (RAW264.7) treated with fucoxanthin inhibited markers of bone resorption and osteoclast differentiation by regulating the expression of some mitogen-activated protein kinases and nuclear factor erythroid 2-related factor 2 (NRF_2_), therefore providing a therapeutic benefit for osteoclast-related diseases such as osteoporosis [92]. Considering the present findings, additional research should evaluate the effects of dietary supplementation with microalgae extract of PT containing fucoxanthin on BMC, BMD, bone resorption, insulin, and related markers in post-menopausal women due to the major physiological and hormonal changes that negatively impact bone health and body composition in this population.

### 5.2. Secondary Outcomes

Our secondary aims were to determine whether fucoxanthin supplementation during an exercise and energy-restricted diet intervention affected adaptations to training, lipid profiles, markers of inflammation and oxidative stress, appetite, and/or perceptions of functional capacity. The rationale was that if fucoxanthin affects REE, lipolysis, and/or markers of inflammation, it may enhance training adaptations, markers of health, and/or perceptions about exercise tolerance. In the present study, participants supplementing their diet with fucoxanthin experienced a greater increase in relative VO_2peak_ after 6 weeks of supplementation (~2.8 mL/kg/min or 10%) and reduction in resting heart rate (~−6.3 beats/min or −8.5%). The increase in aerobic capacity and RHR observed is consistent with our previous research showing this type of exercise program can improve aerobic capacity and decrease resting heart rates in sedentary overweight women initiating an exercise program [36,74,75,76] as well as several meta-analyses [93,94,95] that reported increases in VO_2peak_ in the range of 1.8–7.3 mL/min/kg, depending on the type (aerobic, resistance, interval, etc.) and duration of training. Interestingly, the results are also similar to Hernández et al. [15] who reported that *Arthrospira* (Spirulina maxima) supplementation (4.5 g/day for 12 weeks) combined with aerobic exercise training significantly increased in VO_2peak_ (≈2.5 mL/kg/min) and heart rate at the onset of blood lactate (≈4.2 beats/min) while decreasing RHR (≈2.8 beats/min) and weight loss (≈2.3 kg) in overweight and obese participants [15]. Spirulina maxima is a blue–green marine algae that has also been reported to have antioxidant, anti-inflammatory, and lipolytic properties [96]. The reduction in RHR is particularly noteworthy given its relationship to reducing myocardial oxygen demand and risk to cardiovascular disease [97]. Additional research should explore the role of fucoxanthin in aerobic capacity and cardiovascular risk.

In the present study, there was evidence that fucoxanthin supplementation promoted a more favorable improvement in lipid profiles. In this regard, after 12 weeks of intervention, HDL levels tended to be higher (7.7 mg/dL or 14.5%), and the ratio of LDL-HDL tended to be lower (−0.488 or −21%) with fucoxanthin supplementation. These findings support the findings of Beppu and coworkers [98] who reported that fucoxanthin feeding (2%) increased HDL and reduced hepatic cholesterol content in diabetic/obese mice as well as Woo and colleagues [27] who reported that C57BL/6N mice fed high-fat diets with 0.05 and 0.2% fucoxanthin observed a 20% and 35% increase in HDL cholesterol, respectfully. In human studies, Hitoe et al. [34] reported that fucoxanthin supplementation (1 mg/d for 4 weeks) increased HDL levels by about 3.8% in mildly obese participants. However, HDL levels were not significantly affected in a group consuming 3 mg/d of fucoxanthin. Collectively, the present findings and others support contentions that fucoxanthin supplementation may help increase HDL cholesterol, which is inversely related to cardiovascular risk [16,20]. Additional research should evaluate the impact of fucoxanthin supplementation in middle-aged individuals with elevated lipid levels as well as lipoprotein subfractions and particle size.

In terms of inflammatory and oxidative stress markers, we examined the impact of fucoxanthin supplementation on a panel of inflammatory markers and cytokines. We found evidence that IL-2, IL-6, and TNF-α increased to a greater degree with fucoxanthin supplementation. While these results contrast contentions that fucoxanthin may serve as an anti-inflammatory, participants in the fucoxanthin group saw greater increases in aerobic exercise (steps/day) and resistance training volume. Aerobic exercise increases oxidative stress and resistance-exercise promotes inflammation. Consequently, the higher cytokine and inflammatory response may simply reflect a higher training volume maintained during the study. However, there is also evidence that fucoxanthin may affect mitochondrial function, reactive oxygen species (ROS) production, and PGC1-α activity [99,100]. Aerobic capacity is closely related to mitochondrial activity (e.g., number, function, permeability to ROS, etc.). Improvement in mitochondria activity and function leads to greater efficiency in aerobic energy production and the oxidation of fat as a metabolic fuel. Since it takes more oxygen to oxidize fat, it is plausible that fucoxanthin may have improved mitochondrial function leading to the observed increase in peak aerobic capacity observed. To support this contention, we observed a clinically significant increase in resting fat oxidation (12.1 [10.9, 23.0] %, *p* = 0.031) after 6 weeks of fucoxanthin supplementation. Interestingly, this finding is consistent with the impact that caffeine and green tea extracts have on resting fat oxidation [101,102]. Moreover, this finding suggests the positive impact of microalgae extract of PT supplementation on PGC1-α, which is a central regulator of exercise-induced improvement in mitochondria activities and biogenesis [99,100]. Interestingly, recent studies suggest PGC1-α in bone metabolism, which may support the positive impact of microalgae-based extract of PT on BMC and BMD [103]. PGC1-α has also been reported to mediate exercise-induced angiogenesis, possibly via angiogenic-regulating factors like vascular endothelial growth factor (VEGF) [99,100,104,105,106]. Exercise-induced skeletal muscle angiogenesis is a well-known physiological adaptation to exercise and may thereby lead to improved endurance performance capacity and cardiovascular function [104,107]. Moreover, fucoxanthin has been reported to increase Kelch-like ECH-associated protein 1 (KEAP_1_)-NRF_2_ pathway activity, which participates in regulating angiogenesis and mitochondrial biogenesis with positive interactions with the PGC1-α pathway [105,108,109]. Angiogenic pathways have been reported to induce the release of several cytokines like TNF- α and GMC-SF in the early activation phase [110,111]. Thus, the increase in IL-2, IL-6, TNF-α, and VO_2peak_ observed in the fucoxanthin-supplemented group could also be related to the role of fucoxanthin in these pathways. However, more research is needed to explore the impact of fucoxanthin on inflammatory and oxidative stress markers, mitochondrial function, and cardiovascular adaptations to exercise.

The present study also examined whether fucoxanthin supplementation may affect appetite and appetite-regulating hormones. Interestingly, we found that the FX group participants were able to adhere to the hypoenergetic diet to a greater degree than those in the placebo group. This resulted in a greater reduction in self-reported energy and fat intake. In addition, serum leptin levels were maintained in the FX group while significantly decreasing in the placebo group. Leptin serves to suppress appetite in individuals without leptin resistance and decreases with weight loss in overweight individuals with leptin resistance [14,36,74,75,112]. However, fucoxanthin supplementation has been reported to either not affect or increase leptin levels [18,20,113]. Thus, the supplementation of fucoxanthin during an exercise and weight loss program may have served to better maintain leptin and thereby better suppress appetite. Additional research should investigate whether acute and/or chronic fucoxanthin supplementation affects appetite, regulating hormones, glucose tolerance, and ad libitum food intake.

Finally, we evaluated the effects of fucoxanthin supplementation during an exercise and weight loss program on perceptions of quality of life and functional capacity. The rationale was that if fucoxanthin supplementation improved weight loss and/or training adaptations, participants may perceive an improvement in the ability to perform daily functional activities and ratings of life satisfaction. In this study, we found that those taking fucoxanthin perceived less difficulty bending, kneeling, stooping, and walking several hundred yards, and pain performing daily activities, while feeling more full of life over time. Limitations in exercise capacity, cardiovascular risk factors and bone health, and the perception of physical limitations are well recognized as barriers to engagement with a weight management interventions [114]. Thus, the supplementation of microalgae extracts from PT containing fucoxanthin could optimize the long-term benefits of weight management interventions and adherence to a healthy lifestyle. As we are not aware of any study in humans that reported that fucoxanthin improved perceptions about functional activities and/or quality of life, additional research is needed.

### 5.3. Limitation Considerations

There are several limitations that should be considered when interpreting these findings. First, although statistical significance was observed in several variables, we observed several statistical trends (*p* > 0.05 to *p* < 0.10) with medium to large effect sizes. Consequently, adding more participants to increase statistical power in this study may have yielded significant results. Second, adding a non-exercise and no-diet-intervention control group (with and without fucoxanthin supplementation) would help determine the additive effects of fucoxanthin supplementation with or without exercise and diet intervention as well as whether fucoxanthin supplementation while maintaining an ad libitum diet affects appetite and/or weight loss. Third, this study only examined ingesting one dose of fucoxanthin (4.4 mg/d) for 12 weeks. It is possible that higher doses or multiple doses of FX ingested daily (e.g., ingesting 2.2 mg prior to meals and/or upon retiring) may have had a greater impact on body composition and other health markers. It is also possible that the small amount of omega 3 fatty acids (EPA and DHA) or other naturally occurring compounds in PT may have provided a synergistic effect on fucoxanthin. However, the amount of EPA and DHA found in 220 mg of PT is well below doses reported to have biological activity. Fourth, we only examined the effects of fucoxanthin supplementation while taking part in an exercise and weight loss diet intervention in overweight but otherwise healthy women with normal blood glucose and lipid levels. It is plausible that fucoxanthin supplementation may have greater benefit in men or individuals with glucose intolerance and/or elevated blood lipids. Additionally, while we examined a broad panel of health markers, we did not evaluate markers of all the pathways fucoxanthin has been reported to influence. It is possible that more definitive findings could be observed by analyzing some of these variables. Finally, although the finding that women engaged in exercise and diet intervention while taking fucoxanthin maintained BMC and increased BMD, the difference between the groups was small and additional research is needed to explore this potential relationship.

## 6. Conclusions and Future Directions

Dietary supplementation with microalgae extracts from *Phaeodactylum tricornutum* containing 4.4 mg/d of fucoxanthin for 12 weeks did not promote additional weight loss or more favorable body composition changes in overweight but otherwise healthy females initiating an exercise and diet intervention designed to promote modest weight loss. However, fucoxanthin supplementation preserved bone mass, increased bone density, and saw greater improvements in walking steps/day, resting heart rate, aerobic capacity, blood lipid profiles, adherence to diet goals, functional activity tolerance, pain, and measures of quality of life. Consequently, there appears to be some benefit to the dietary supplementation of fucoxanthin while participating in a diet and exercise program. More research should evaluate the potential health benefits of fucoxanthin supplementation in sedentary and active men and women with and without diet intervention. Additionally, work should also evaluate the effects of fucoxanthin supplementation on bone turnover, BMC, and BMD in post-menopausal women and individuals with glucose intolerance, diabetes mellitus, high blood lipids, functional capacity limitations, and perceptions of limited quality of life.

## Data Availability

Data and statistical analyses are available for non-commercial scientific inquiry and/or educational if request and use does not violate IRB restrictions and/or research agreement terms.

## Supplementary Materials

The following supplemental materials are available online at https://www.mdpi.com/article/10.3390/nu16070990/s1, Table S1: Participant Demographic Data, Table S2: Energy and Macronutrient Intake, Table S3: Resistance and Endurance Training Volume, Table S4: Body Composition and Anthropometric Measurements, Table S5: Bone Mineral Content, Area, and Density, Table S6: Resting Energy Expenditure, Table S7: Aerobic Capacity, Table S8: Muscular Strength and Endurance, Table S9: Complete Blood Counts, Table S10: Serum Comprehensive Metabolic Analysis, Table S11: Glucose Homeostasis and Insulin Sensitivity Analysis, Table S12: Leptin and Blood Lipid Analysis, Table S13: Cytokine and Inflammatory Marker Analysis; Table S14: Quality of Life, Table S15: Resting Hemodynamic Data, Table S16: Frequency and Severity of Side Effects.
